# Unraveling the Effects of Biochemical Drivers on the Bacterial Communities and Volatile Profiles in Refrigerated Sturgeon Filets at 4°C

**DOI:** 10.3389/fmicb.2022.849236

**Published:** 2022-03-30

**Authors:** Chunming Tan, Mengyuan Xiao, Ruiyun Wu, Pinglan Li, Nan Shang

**Affiliations:** ^1^Beijing Laboratory for Food Quality and Safety, College of Food Science and Nutritional Engineering, China Agricultural University, Beijing, China; ^2^College of Engineering, China Agricultural University, Beijing, China; ^3^Key Laboratory of Precision Nutrition and Food Quality, Department of Nutrition and Health, China Agricultural University, Beijing, China

**Keywords:** spoilage bacteria, microbial communities, volatile organic compounds, GC-IMS, high-throughput sequencing

## Abstract

Spoilage bacteria seriously influence the flavor and quality of fish meat. In this study, we investigated the quality characteristics, bacterial community, and volatile profiles of refrigerated (4°C) sturgeon filets during 10-day storage. On day 10, the refrigerated samples showed the lowest bacterial diversity and the largest difference in microbiota and biochemistry. The dominant genera in the fresh samples were *Macrococcus*, *Acinetobacter*, *Moraxella*, *Brucella*, and *Pseudomonas*, while the dominant bacteria changed into *Acinetobacter*, *Carnobacterium*, *Macrococcus*, *Pseudomonas*, and *Psychrobacter* at the end of storage. Our results suggest that these dominant taxa contribute to the spoilage of the refrigerated sturgeon filets. Meanwhile, during the storage, total viable counts, total volatile basic nitrogen, thiobarbituric acid-reactive substances, and trichloroacetic acid-soluble peptide significantly increased (*P* < 0.05), while the sensory score decreased steadily. Additionally, the ATP-related compounds and the *K*-value showed similarly increasing trends. The shelf-life of the refrigerated sturgeon filets was less than 8 days. The gas chromatography–ion mobility spectrometry results suggest that hexanal, ethyl acetate, ethanol, butanal, 1-propanol, isopentyl alcohol, 2-pentanone, 2-heptanone, ethyl propanoate, and propyl sulfide are potential chemical spoilage markers. The predicted metabolic pathways indicated an abundant carbohydrate metabolism and amino metabolism in the refrigerated sturgeon filets. This study provides insight into the determinants of sturgeon shelf-life and the spoilage process involved in refrigerated fish.

## Introduction

Sturgeon is one of the most important and precious fish resources due to their palatability and nutritional value. In recent years, sturgeon aquaculture has developed rapidly, especially in China, whose production accounts for 85% of the world’s total (Chen et al., 2020). However, the storage and preservation of sturgeon meat has become a big challenge for people due to its high moisture content and inevitable microbial activity. The microbial metabolic activities cause fish spoilage by affecting the pH, the degradation of nutrients, the generation of total volatile basic nitrogen (TVB-N), the production of thiobarbituric acid-reactive substances (TBARS) and volatile organic compounds (VOCs), etc. (Zhang et al., 2015; Liu et al., 2018a; Li et al., 2020a). However, there is limited research focusing on the biochemical and quality changes of sturgeon filets during refrigerated storage. Although Zhang Y. et al. (2021), Zhang Z. et al. (2021) reported on the microbial changes of sturgeon being stored at ice temperature, only the specific spoilage organisms (SSOs) were evaluated. The effects of biochemical drivers on the bacterial communities and volatile profiles of refrigerated sturgeon filets at 4°C are still unknown. In addition, the microbiota in aquatic products was altered dramatically with storage time and many other factors like environment, aquaculture species, processing operation, etc. (Parlapani et al., 2018; Hauptmann et al., 2020). Hybrid sturgeon, a popular commercial fish, and the sales of refrigerated sturgeon filets are growing because of its convenience for subsequent cooking. Therefore, monitoring the development of the bacterial community and quality characteristics of refrigerated sturgeon filets during storage is important to identify the predominant spoilage bacteria and thus prevent sturgeon meat putrefaction and extend the shelf-life.

Fish is a highly perishable food, and its spoilage is a consequence of complex processes involving physical, chemical, and biological mechanisms, which result in food quality and human health issues. Along with microbial growth, the flavor and the quality of fish greatly change with protein degradation and nucleotide catabolism. Gui et al. (2014) reported that more than 90% of the nucleotides in fish muscle were purine derivatives resulting from the catabolism of adenosine triphosphate (ATP), and the fish freshness could be evaluated by calculating the ATP and related compounds as well as the *K*-value—for example, intermediate nucleotides like inosine monophosphate (IMP) and hypoxanthine (Hx) act as a flavor enhancer and a bitter compound, respectively (Hong et al., 2017). Protein also accounts for 15–22% of fish muscle, and its degradation may cause muscle softening, discoloring, water loss, and flavor changes (Li et al., 2020a). The volatile flavors in fish are mainly generated by enzymatic reactions, lipid oxidation, microbial activity as well as the conditions of processing and storage (Li et al., 2018c). Among them, the VOCs associated with bacterial metabolites include alcohols, aldehydes, esters, ketones, sulfur compounds, and other molecules (Parlapani et al., 2017). Lv et al. (2020) reported that there is an inter-relationship between the changes of VOCs during the fermentation of shrimp paste and the bacterial community succession. Jia et al. (2019) indicated that the VOCs of silver carp during chill-storage showed a good correlation with the growth of potential spoilage-dominant bacteria. Therefore, some VOCs can be used for the spoilage assessment of fish and fish products during storage.

Previous studies have shown that fish spoilage is mainly caused by a group of microorganisms. They are associated with biochemical changes in fish (Kuuliala et al., 2018; Jia et al., 2019). However, only a small quantity of microbial communities contributes to the spoilage and causes undesirable changes (Gram et al., 2002). Pennacchia et al. (2011) reported some specific spoilage organisms presenting in fresh fish, which can eventually become dominant spoilage microorganisms. Therefore, it is valuable to reveal the dominant bacteria at different storage stages and to analyze the relationship between microbial communities and quality changes in fish or fish products. Recently, high-throughput sequencing based on the 16S rRNA gene has been applied to explore the diversity and composition of microorganisms, which provides a powerful tool to comprehensively reveal the dynamics of spoilage bacteria and analyze the correlations between microbial metabolic pathways and spoilage bacteria (Li et al., 2019). The objectives of this study are to explore the biochemical changes including protein degradation, nucleotide catabolism, lipid oxidation, and volatile production and to investigate the microbial community dynamics in sturgeon filets during refrigerated storage.

## Materials and Methods

### Sampling Preparation and Storage

Cultured hybrid sturgeon (*Acipenser schrenckii* ♂× *Acipenser baeri*♀) with an average weight and length of 4,643 ± 217.8 g and 76.2 ± 8.4 cm, respectively, were obtained from the Sturgeon Farm of Tianyuan Fishing Port (Huairou, Beijing, China) and immediately transported to the laboratory (less than 3 h). Subsequently, the sturgeons were stunned by tapping on the head, scaled, gutted, and washed with cold sterile water immediately, after which they were fileted (weighing 161.7 ± 9.47 g each, *n* = 36). All filets were drained on sterile gauze for 15 min and then placed in CLEANWRAP fresh-keeping bags [Cleanwrap, Korea; oxygen/water permission rate was 13,960 cm^3^/(m^2^ × d × atm), 25 cm × 35 cm] and stored at 4 ± 1°C. Three filets were selected randomly for analysis on days 0, 2, 4, 6, 8, and 10. All parameters were determined in triplicate except for sensory evaluation, which was carried out six times.

### Sensory Analysis

The sensory properties, including appearance, texture, and odor, were assessed according to the previous methods (Amerine et al., 1965 and Huang et al., 2018), with some modifications. Six trained and experienced postgraduate students formed the assessment group. They were familiar with the sensory characteristics and evaluation standards of fish species. A score of 8.0–9.0 indicated good quality, 6.0–7.9 indicated acceptable quality, 4.0–5.9 indicated spoiled quality, and 1.0–3.9 denoted complete spoilage, which meant that the filet had a dull color, a strong stench, and a flabby texture.

### Determination of pH and Total Volatile Basic Nitrogen

The pH and TVB-N of the filets was determined as described by Gui et al. (2014). The TVB-N value was expressed as milligram per 100 g of flesh.

### Determination of Adenosine Triphosphate-Related Compounds and K-Value

The ATP-related compounds were extracted and analyzed according to Huang et al. (2018), with some modifications. In total, 5 g fish meat was homogenized with 10 ml 0.6 M cold perchloric acid (PCA) solution for 1 min and centrifuged (10,000 *g* for 10 min). The precipitate was washed twice with 0.6 M PCA, and the supernatant was combined. Then, all supernatants were obtained to adjust the pH to 6.50 ± 0.05 with NaOH solution, followed by centrifugation at 10,000 *g* for 15 min, and the precipitate was washed with 0.6 M neutralized PCA (pH 6.5). Then, the supernatant was combined and made up to 50 ml using neutralized PCA and stored at −20°C for further analysis. All the above-mentioned reagents need to be pre-cooled, and the entire operation is performed on crushed ice.

The ATP-related compounds were analyzed by high-performance liquid chromatography (Shimadzu LC-20 series, Kyoto, Japan) that was equipped with SPD-20A (M) detector and a Shim-pack VP-ODS column (2.0 mm ID × 250 mm × 5 μm). The mobile phase was 0.05 M phosphate buffer (pH 6.8), and the flow rate was 0.2 ml/min. The injection volume was 20 μl, and the detection wavelength was 254 nm. ATP-related compounds were determined and calculated based on the retention time and peak area compared with standard solutions, and *K*-value was defined by the following equation:


K-value(%)=[(HxR+Hx)/(ATP+ADP+AMP+IMP+HxR+Hx)]×100

### Analysis of Protein Degradation and TCA-Soluble Peptides

The proteins of sturgeon flesh were extracted and detected according to the method of BCA Protein Assay Kit (Nanjing Jiancheng Bioengineering Institute, Jiangsu, China). Sodium dodecyl sulfate–polyacrylamide gel electrophoresis (SDS-PAGE) (Bio-Rad, United States) was performed using 12% separating gel with 4% stacking gel for proteins. Then, 15 μl of protein sample was loaded onto the gel, and electrophoresis was performed at a constant voltage of 125 V for 35 min. After separation, the gels were immersed successively in stainer (0.25% Coomassie brilliant blue R-250, 10% acetic acid, and 50% ethanol) for 1.5 h and in destainer (8% acetic acid and 25% ethanol) for 2 to 3 h. The concentration of trichloroacetic acid (TCA)-soluble peptides of sturgeon flesh was measured by the method of Jia et al. (2019). Briefly, 3 g fish muscle was homogenized with 27 ml of 5% (w/v) cold TCA and stored in crushed ice for 30 min. The obtained homogenate was centrifuged at 8,000 *g* for 5 min at 4°C. The supernatant was measured by the Lowry method and expressed as micromole of tyrosine per gram.

### Determination of Thiobarbituric Acid-Reactive Substances

TBARS was measured using the malondialdehyde (MDA) Assay Kit (Beijing Solarbio Science and Technology Co., Ltd., Beijing, China) to determine lipid peroxidation, and the result was expressed as milligrams of MDA per kilogram of flesh.

### Analysis of Volatile Organic Compounds by Gas Chromatography–Ion Mobility Spectrometry

An analysis of the VOCs of fish muscle was performed on a gas chromatography–ion mobility spectrometry (GC–IMS) system (FlavourSpec^®^, Gesellschaft für Analytische Sensorsysteme mbH, Dortmund, Germany). The extraction and determination of VOCs from fish were carried out according to the method of Li et al. (2020a). Briefly, 2 g of minced muscle of each sample was placed at 40°C for 20 min in a 20-ml headspace vial sealed with crimp caps. Then, 500 μl of the headspace was injected into the heated injector (shaking with heating at 85°C, 500 r/min) of the GC–IMS instrument by a heated syringe (85°C). The VOCs were separated and analyzed by GC–IMS as follows: column temperature at 60°C and carrier gas flow rate at 2 ml/min for 10 min, 10 ml/min for 10 min, and 100 ml/min for 5 min. After being eluted in the isothermal mode, the analytes were driven into the ionization chamber before detection by the IMS. The volatile constituents were ionized and driven to a 9.8-cm drift tube (5 kV constant voltage, 45°C) by 150 ml/min nitrogen, which was separated again and detected.

### Microbiological Analyses

#### Total Viable Counts

The total viable counts (TVC) of the filets were determined according to the method of Shen et al. (2020). In total, 10 g of fish sample was aseptically weighed and homogenized with 90 ml sterile 0.85% NaCl solution for 60 s in a sterile homogeneous bag, and then 1 ml of supernatant was taken for gradient dilution. A suitable dilution samples (0.1 ml) was spread on plate count agar (PCA, Hai Bo Biological Technology Co., Ltd., Qingdao, China) and incubated at 30 ± 1°C for 48 h. All tests were carried out six times in parallel, and the microbiological number was expressed as log colony-forming unit (CFU)/g.

#### High-Throughput Sequencing

Total microbial genomic DNA extraction was conducted, and the 16S rRNA V3–V4 region was amplified as described by Liu et al. (2018b). The PCR products were detected by 2% agarose gel electrophoresis, and the amplicons were purified and quantified using the AxyPrep DNA Gel Extraction Kit (Axygen, CA, United States) and QuantiFluor*™*-ST (Promega, United States), respectively. A high-quality amplicon library for high-throughput pyrosequencing was constructed using the TruSeq^®^ DNA Library Prep Kit (Illumina Inc., San Diego, CA, United States). The paired-end sequencing (2 × 300 bp) was carried out by the Illumina MiSeq platform (Illumina Inc., San Diego, CA, United States). All obtained raw sequence datasets in this work have been uploaded to the NCBI Sequence Read Archive with BioProject PRJNA786422.

The quality-filtered sequences were clustered into operational taxonomic units (OTUs) with 97% similarity threshold using UPARSE (version 7.0.1^1^), and chimeric sequences were identified and removed using Uchime software (version 8.1). The taxonomy of each 16S rRNA gene sequence was analyzed by RDP Classifier (version 2.11^2^) against the Silva 16S rRNA database (Release 132^3^) using 70% confidence. The alpha diversity (Simpson, Shannon, ACE, Chao1, and Goods coverage indexes) was determined using Mothur (version 1.30^4^) with 97% similarity. The visualized principal coordinate analysis (PCoA) of beta diversity was calculated using the Bray–Curtis distance matrix to reflect the differences of the bacterial community profiles in the samples. In addition, PICRUSt2 predicted the functional profiling of the metagenome based on bacterial amplicon sequencing profiles (Douglas et al., 2020). The functional annotation of the predicted features was carried out based on the Kyoto Encyclopedia of Genes and Genomes database.

### Statistical Analysis

The data were analyzed by SPSS 21.0 software (SPSS Inc., Chicago, IL, United States). One-way analysis of variance (ANOVA) with Duncan’s test was used to determine the significance among various treatments. A value of *P* < 0.05 was considered significantly different. The Spearman correlation coefficient was calculated among different samples to analyze their relationships.

## Results

### Quality and Shelf-Life

**FIGURE 1:**
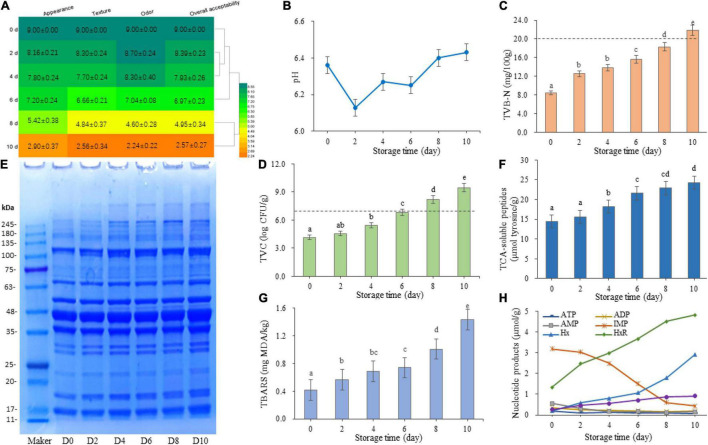
Changes in sensory index **(A)**, pH value **(B)**, total volatile basic nitrogen **(C)**, total viable counts **(D)**, protein degradation **(E)**, trichloroacetic acid-soluble peptides **(F)**, thiobarbituric acid reactive substances **(G)**, and nucleotide degradation products content and *K*-value **(H)** of sturgeon filets during storage at 4°C. The sensory scores were processed using Heml 1.0.3.7. Different lowercase letters indicate significant differences (*P* < 0.05) in the samples.

To monitor quality changes in the refrigerated sturgeon filets, the sensory scores (appearance, odor, and texture) of all samples were evaluated (Figure 1A). The initial sensory index was tested in the completely fresh state, and with the extension of storage time, the sensory scores decreased significantly (*P* < 0.05). Generally, fish meat is considered unacceptable for human consumption when the overall score of sensory evaluation was below 5 (Huang et al., 2018). Therefore, based on our sensory evaluation, the shelf-life of sturgeon filets was less than 8 days. On day 8, the texture changed from tight to soft, accompanied by plenty of liquid, and an unpleasant odor was formed.

Furthermore, the pH and TVB-N values of the samples during storage were determined. As shown in Figures 1A,B, although the pH value exhibited fluctuations during the first 6 days, the pH value of the samples increased significantly after 8 days and showed an increasing trend. The content of TVB-N is another direct indicator of meat quality and used to evaluate the microbial spoilage of fish. As shown in Figure 1C, the TVB-N production in sturgeon filets displayed an obvious upward trend (*P* < 0.05) during refrigerated storage. The results showed that the endogenous enzymes were with intense activity at day 0, and the amino acid degradation by microorganisms happened after 6 days. After 10 days of storage, the TVB-N content reached 21.84 ± 0.11 mg/100 g, which is higher than the limit (20 mg/100 g), but the content was acceptable within 8 days.

The change of TVC in refrigerated sturgeon filets during storage was monitored on PCA media (Figure 1D). The initial TVC value of the samples was 4.17 ± 0.14 log CFU/g and significantly increased (*P* < 0.05) with storage time, especially after 4 days of storage (from 5.43 ± 0.34 to 9.47 ± 0.12 log CFU/g). According to the International Commission of Microbiological Specializations on Food (ICMSF, 1986), the initial TVC and the upper tolerable limit of fresh fish are 2–5 log CFU/g and 7 log CFU/g, respectively. The refrigerated sturgeon filets had a relatively good quality up to day 8, with a TVC of 8.22 ± 0.40 log CFU/g, which was consistent with the result of our sensory scores. The above-mentioned quality indicators showed that the shelf-life of refrigerated sturgeon filets was within 8 days.

### Proteolytic Activities and Trichloroacetic Acid-Soluble Peptide Contents

Protein changes in sturgeon filets were observed during storage, as shown in Figure 1E. The band intensity at around 17, 18.5, 23, 70, and 120 kDa was increased, and the bands at around 50, 150, and 260 kDa showed up. The other protein fractions remained relatively stable during storage. The concentration of TCA-soluble peptides is an important biochemical indictor to monitor protein degradation, which is mainly produced during the breakdown of protein. As shown in Figure 1F, the initial concentration of TCA-soluble peptides on day 0 was 14.39 ± 1.25 μmol tyrosine/g sample and significantly increased (*P* < 0.05) after 2 days of storage. The TCA-soluble peptides reached 24.30 ± 0.74 μmol tyrosine/g sample on day 10. The increase in soluble peptides corresponded to the growth trend of bacteria (Figure 1D), suggesting that protein degradation in refrigerated sturgeon filets is related to bacterial activities.

### Lipid Oxidation

The TBARS value was selected as the chemical indicator of lipid oxidation by measuring the content of MDA. MDA is formed by the reaction of polyunsaturated fatty acids with oxygen (Fernández et al., 1997). The initial TBARS value was 0.302 ± 0.002 mg MDA/kg and showed a significant increase (*P* < 0.05) with storage time, especially after 6 days (Figure 1G). On day 8, the TBARS value of sturgeon filets reached 1.008 ± 0.005 mg MDA/kg. However, 1 mg MDA/kg is usually considered as the limit for normal odor (Huang et al., 2018). In this study, a higher TBARS value was obtained in the samples after 10 days of storage (1.431 ± 0.003 mg MDA/kg), which indicated high oxidative rancidity.

### Catabolism of Nucleotides

The changes of ATP and ATP-related compounds of refrigerated sturgeon filets during storage are shown in Figure 1H. The initial concentrations of ATP, ADP, AMP, IMP, HxR, and Hx were 0.19, 0.35, 0.56, 3.18, 1.32, and 0.20 μmol/g, respectively. With the increase of storage time, ATP rapidly degraded to IMP and eventually converted to Hx. IMP is the main nucleotide present in sturgeon fish post-mortem, which was broken down from AMP and sequentially catabolized to HxR and Hx. In this study, IMP degraded rapidly after 2 days of storage (*P* < 0.05), reducing the flesh quality. The Hx content of flesh was very low on day 0, but with the degradation of IMP, Hx increased to 2.92 μmol/g on day 10. The *K*-value is defined as the ratio of non-phosphorylated ATP breakdown products to the total ATP breakdown products, which is commonly used to evaluate the freshness of aquatic products. The *K*-value gradually increased with storage time (Figure 1H), which showed a significant increase (*P* < 0.05) during 0–8 days and then tend to be stable. According to the quality and shelf-life indicators, the rejection level of the *K*-value was approximately 75% for refrigerated sturgeon filets.

### Volatile Organic Compounds

**FIGURE 2:**
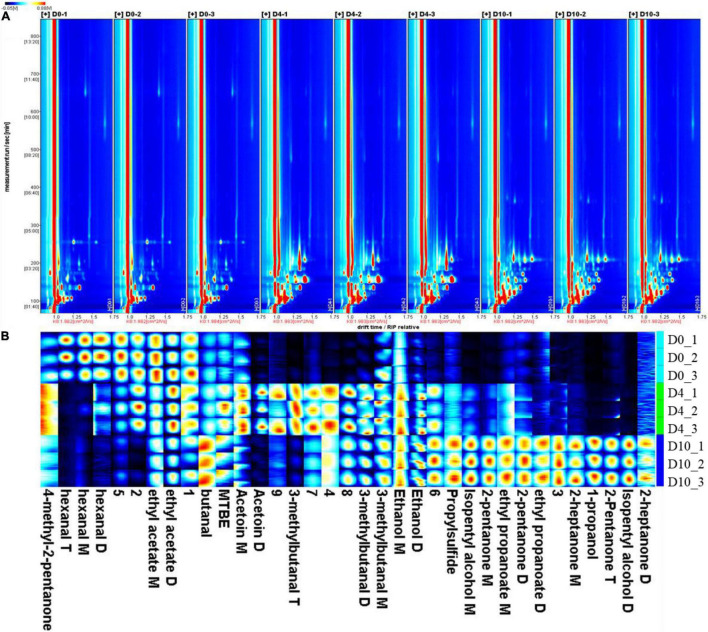
The volatile organic compounds (VOCs) identified in sturgeon filets on days 0, 4, and 10 during storage at 4°C. **(A)** Topographical plots corresponding to signals detected in samples and **(B)** fingerprint comparison of VOCs in samples determined by gas chromatography–ion mobility spectrometry.

The VOCs in refrigerated sturgeon filets were detected on days 0, 4, and 10 by GC–IMS. A total of 20 VOCs were identified, which mainly include alcohols, aldehydes, ketones, esters, and sulfur compounds (Table 1). The VOCs were present in different polymerization degrees, including monomers, dimers, and trimers, depending upon their concentrations. These products exhibited similar retention times but different drift times. As shown in Figure 2A, the spot intensity in the VOCs profile reflects the compound changes (increased, decreased, disappeared, and fluctuated) during storage. With the extension of storage time, the number of volatile substances in fish meat was increased. The PCA analysis (Supplementary Figure 1) also indicated that there was a significant difference between fresh sturgeon filets and refrigerated sturgeon filets, which was time dependent. As shown in Figure 2B, each column represented a signal peak of one volatile compound in the fingerprint, and the brighter spot indicated the higher concentration of the volatile compound. The signal intensities of hexanal, ethyl acetate, and 4-methyl-2-pentanone gradually decreased with storage time. Among them, 4-methyl-2-pentanone was only detected in fresh fish filets, and hexanal disappeared at 10 days. Meanwhile, ethanol, butanal, 1-propanol, isopentyl alcohol, 2-pentanone, 2-heptanone, ethyl propanoate, and propyl sulfide gradually increased with storage time as well, and all of them except ethanol were not detected in fresh filets. Especially 2-pentanone, 2-heptanone, 1-propanol, and ethyl propanoate were only detected at day 10.

**TABLE 1:** The volatile organic compounds in refrigerated sturgeon filet samples detected by gas chromatography–ion mobility spectrometry.

Compounds	CAS number	Formula	Molecular weight	Retention index	Retention time (s)	Drift time (ms)	Comment
**Alcohols**							
Ethanol	C64175	C_2_H_6_O	46.1	503.3	94.569	1.0408	Monomer
Ethanol	C64175	C_2_H_6_O	46.1	520.0	101.569	1.1279	Dimer
1-Propanol	C71238	C_3_H_8_O	60.1	569.0	122.146	1.1884	Monomer
1-Propanol	C71238	C_3_H_8_O	60.1	571.5	123.224	1.2533	Dimer
MTBE	C1634044	C_5_H_12_O	88.1	565.3	120.607	1.3509	Monomer
Isopentyl alcohol M	C123513	C_5_H_12_O	88.1	738.3	212.441	1.2416	Monomer
Isopentyl alcohol D	C123513	C_5_H_12_O	88.1	735.9	210.555	1.4894	Dimer
**Aldehydes**							
Butanal	C123728	C_4_H_8_O	72.1	592.1	131.850	1.2830	Monomer
3-Methylbutanal	C590863	C_5_H_10_O	86.1	647.2	155.014	1.1689	Monomer
3-Methylbutanal	C590863	C_5_H_10_O	86.1	646.0	154.492	1.2784	Dimer
3-Methylbutanal	C590863	C_5_H_10_O	86.1	649.4	155.916	1.4073	Trimer
Hexanal	C66251	C_6_H_12_O	100.2	790.6	256.381	1.2590	Monomer
Hexanal	C66251	C_6_H_12_O	100.2	788.9	254.571	1.3581	Dimer
Hexanal	C66251	C_6_H_12_O	100.2	789.6	255.303	1.5540	Trimer
**Ketones**							
Acetoin	C513860	C_4_H_8_O_2_	88.1	723.2	200.307	1.0604	Monomer
Acetoin	C513860	C_4_H_8_O_2_	88.1	713.9	192.785	1.3295	Dimer
2-Pentanone	C107879	C_5_H_10_O	86.1	688.0	172.132	1.1199	Monomer
2-Pentanone	C107879	C_5_H_10_O	86.1	685.7	171.204	1.2226	Dimer
2-Pentanone	C107879	C_5_H_10_O	86.1	686.3	171.424	1.3686	Trimer
4-Methyl-2-pentanone	C108101	C_6_H_12_O	100.2	731.0	206.598	1.1758	Monomer
2-Heptanone	C110430	C_7_H_14_O	114.2	890.4	363.986	1.2608	Monomer
2-Heptanone	C110430	C_7_H_14_O	114.2	889.4	362.914	1.6245	Dimer
**Esters**							
Ethyl acetate	C141786	C_4_H_8_O_2_	88.1	602.6	136.279	1.0954	Monomer
Ethyl acetate	C141786	C_4_H_8_O_2_	88.1	603.1	136.505	1.3370	Dimer
Ethyl propanoate	C105373	C_5_H_10_O_2_	102.1	704.8	185.460	1.1507	Monomer
Ethyl propanoate	C105373	C_5_H_10_O_2_	102.1	704.5	185.205	1.4526	Dimer
**S-containing compounds**							
Propyl sulfide	C111477	C_6_H_14_S	118.2	896.4	373.44	1.1591	Monomer

### Microbiological Analyses

#### Richness and Diversity of Microbial Community

A total of 417,581 high-quality effective sequences were obtained by merging and filtering raw reads from each sample, with an effective ratio of 93.85–98.92%, and the sequence length ranged from 251 to 540 bp (Supplementary Table 1). With a minimal similarity threshold of 97%, the effective sequences were clustered into 332 OTUs (Supplementary Table 2). The alpha-diversity indexes are shown in Supplementary Table 2. Good’s coverage was at least 99.9% for all samples, indicating that almost all bacteria in the sturgeon samples were detected. At the end of storage, the values of Chao 1, ACE, and Shannon indexes were lower than the initial values (day 0), while the Simpson indexes were higher. Chao1 and ACE indexes represent the community richness, and Shannon and Simpson indexes represent the community diversity (Li et al., 2018a). These results indicated that the microbiome richness and diversity decreased during storage, suggesting that some bacteria became dominant in the samples.

**FIGURE 3:**
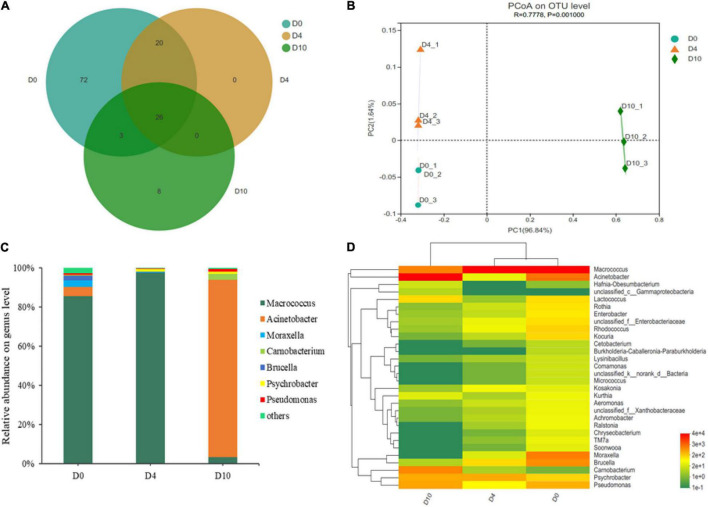
The Venn diagram shows the unique and shared bacterial population **(A)**, principal coordinate analysis of the bacterial community **(B)**, dynamics in relative abundance (%) of bacterial community at genus level **(C)**, and a heat map analysis of microbiota at the genus level **(D)** in sturgeon filets during storage at 4°C.

A Venn diagram was constructed to investigate the similarities and differences between different samples. As shown in Figure 3A, 26 bacterial genera were classified as core genera. The PCoA of all samples was conducted to reveal the differences in bacterial community according to the relative abundance of OTUs (Figure 3B). The samples were not clustered together at different storage times, indicating that the bacterial community changed greatly and the effect of time was significant to the bacterial community.

#### Composition of Microbial Community

In order to investigate the succession of microbial communities of sturgeon filets during refrigerated storage, 16S rRNA gene sequences were classified based on phyla and genera. Firmicutes and Proteobacteria were the major bacterial phyla identified in all samples. On day 10, the percentage of Proteobacteria increased to 93.25%, while the proportion of Firmicutes decreased to 6.74% (Supplementary Figure 2). At the genus level, the community dynamics of 126 bacterial OTUs from all samples were evaluated. *Macrococcus* (85.47%), *Acinetobacter* (4.76%), and *Moraxella* (3.41%) comprised the top three genera at the initial storage time, while *Macrococcus* (97.43%) and *Acinetobacter* (90.54%) became the most abundant genus on days 4 and 10, respectively (Figure 3C). These results indicated that the microbial composition changed dynamically in refrigerated sturgeon filets during storage.

Simultaneously, a hierarchically clustered heat map of the top 30 genera was constructed to analyze and compare the composition and dynamics of microbial communities (Figure 3D). The redder and the greener color illustrated the higher and the lower relative abundance, respectively. As shown in Figure 3D, the microbiota on day 0 showed greater bacterial diversity and was more abundant than those on day 10, indicating that the microbial composition dramatically changed. *Macrococcus*, *Acinetobacter*, *Moraxella*, *Brucella*, and *Pseudomonas* were the dominant genera in the fresh samples, but at the end of storage, the dominant bacteria were changed to *Acinetobacter*, *Carnobacterium*, *Macrococcus*, *Pseudomonas*, and *Psychrobacter*. In this study, the proportions of *Acinetobacter*, *Pseudomonas*, and *Aeromonas* on day 10 were increased to 91–93% compared with 6–8% initially (Figure 3C). In addition, *Kocuria*, *Rhodococcus*, *Enterobacter*, *Rothia*, *Soonwooa*, and some other genera that were identified at the initial time were decreased or even disappeared finally, indicating that they were less competitive compared with those predominant bacteria during refrigerated storage.

#### Functional Properties of the Bacterial Community

**FIGURE 4:**
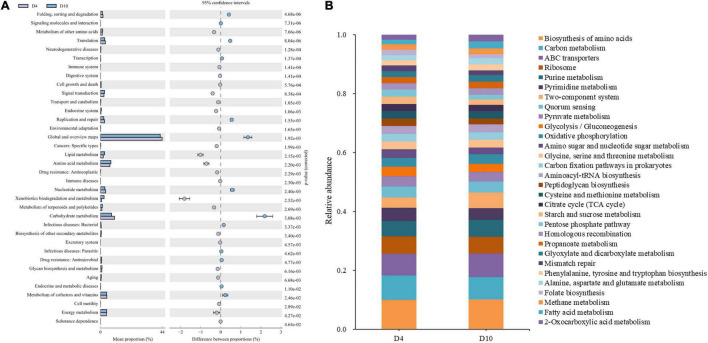
Functional properties that are related to microbial metabolism in the samples. **(A)** Comparison of bacterial metabolic pathways in refrigerated (4°C) sturgeon filets between days 4 and 10. **(B)** Relative abundance of the bacterial three-level metabolic pathways in the top 30. Metabolic inference based on the 16S rRNA sequence.

Microbial metabolism is the most important factor in the spoilage of aquatic products. Thus, metabolic pathways in microorganisms can provide further insight into the sturgeon flesh spoilage process. As shown in Supplementary Figure 3, the samples on days 0 and 4 showed similar metabolic abundance, indicating that the microorganisms associated with spoilage metabolism did not change significantly after 4 days of refrigerated storage. Compared with days 4 and 10 (Figure 4A), the abundance of genes associated with carbohydrate metabolism, nucleotide metabolism, and metabolism of cofactors and vitamins decreased significantly on day 10, but amino acid metabolism, lipid metabolism, energy metabolism, and signal transduction significantly increased. The results indicated that spoilage microorganisms used the nutrients to grow by degrading proteins and carbohydrates and then promoted its spoilage metabolic activities (such as amino acid metabolism, which was associated with spoilage metabolites). Furthermore, the biosynthesis of secondary metabolites and the intracellular secretion and vesicular transport increased significantly at the end of storage (Figure 4A and Supplementary Figure 3), which also indicated that, in the later period of storage, the microorganisms mainly produced metabolites.

The three-level pathway of microbial metabolism in refrigerated sturgeon filets during storage on days 4 and 10 is shown in Figure 4B. The abundance of metabolic pathways associated with biogenic amines and sulfide formation such as phenylalanine, tyrosine, and tryptophan biosynthesis metabolism was higher on day 10 than on day 4, which was related to *Pseudomonas* and *Enterobacteriaceae* (Mazhar et al., 2020). The metabolic pathways associated with the formation of volatile compounds (alcohols, aldehydes, ketones, esters, etc.) were relatively abundant on days 4 and 10, such as pyruvate metabolism. The abundance of propanoate metabolism, glyoxylate and dicarboxylate metabolism, and fatty acid metabolism, which were related to the formation of volatile amines, was higher on day 10. These metabolic pathways further indicated that the microorganisms in the later period of storage were mainly producing spoilage metabolites. However, there was no regular change found between the metabolic pathways and the dominant bacteria on days 4 and 10. Therefore, it is necessary to find out the correlation between bacteria and its metabolism.

### Analysis of the Correlation Among Microbiota, Biochemical Changes, and Metabolic Pathways

**FIGURE 5:**
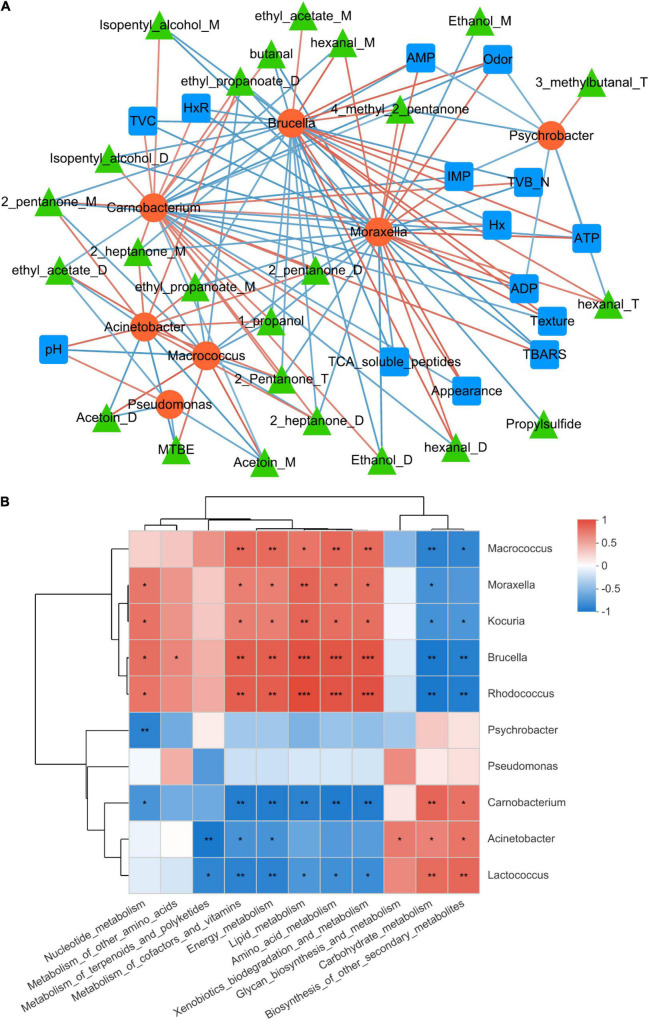
Network analysis of the correlation among microbiota, biochemical changes, and metabolic pathways. **(A)** Correlation between the dominant bacteria and biochemical changes. **(B)** Correlation between the dominant bacteria and metabolic pathways. The microbiota was considered at the operational taxonomic unit level, and statistically significant Pearson correlations were calculated among the sturgeon samples. A connection stands for a significant (*P* < 0.05) and positive (Pearson correlation > |0.7|) correlation. *p < 0.05, **p < 0.01, ***p < 0.001.

The correlation between microbiota and biochemical changes in refrigerated sturgeon filets was analyzed by Pearson correlation analysis (PCA) (Figure 5A), and the detailed values of Pearson correlation coefficients are shown in Supplementary Table 3. Dominant bacteria (relative abundance > 1%) such as *Carnobacterium*, *Moraxella*, *Brucella*, *Macrococcus*, and *Acinetobacter* showed a strong correlation with biochemical changes. *Acinetobacter* had the highest abundance at the end of storage, which positively correlated with pH and the production of VOCs like 1-propanol, 2-pentanone, 2-pentanone, 2-pentanone, ethyl propanoate, ethyl propanoate, 2-heptanone, and 2-heptanone. *Carnobacterium* showed a strong correlation with 26 spoilage metabolites, including TVB-N, TBARS, and VOCs, and negatively correlated with sensory index such as appearance, texture, and odor. In addition, *Moraxella* and *Brucella* had a strong positive correlation with IMP, ATP, ADP, and AMP, while *Carnobacterium* and *Psychrobacter* showed a negative correlation with these ATP-related compounds.

Furthermore, the correlation between dominant bacteria and metabolic pathways in sturgeon filets was analyzed, as shown in Figure 5B. *Macrococcus*, *Brucella*, *Rhodococcus*, *Moraxella*, and *Kocuria* were associated with high levels of carbohydrate metabolism, which showed that these four bacteria had a strong capacity of utilizing carbohydrate. *Acinetobacter* and *Pseudomonas* showed a strong correlation with lipid metabolism, energy metabolism, and secondary metabolites as well as intracellular secretion and vesicular transport. *Acinetobacter* showed the most pronounced positive correlation with lipid metabolism (*R* = 0.88) and secondary metabolite biosynthesis (*R* = 0.88), which is consistent with the significant increase of lipid metabolism and secondary metabolites in microorganisms shown in Figure 4A. However, *Pseudomonas* in the samples showed the most pronounced positive correlation with energy metabolism (*R* = 0.85), which was also consistent with the abundance of reads associated with these metabolic processes on day 10.

## Discussion

Fish spoilage is a consequence of complex processes and mainly caused by a group of microorganisms, which is associated with biochemical changes and microbial community. Previous studies have shown that only a small quantity of microbial communities present in fresh fish can eventually become dominant spoilage microorganisms (Pennacchia et al., 2011). In order to reveal the relationship between dominant bacteria and quality changes, we explored the biochemical changes and microbial community dynamics of sturgeon filets during refrigerated storage, including protein degradation, nucleotide catabolism, lipid oxidation, volatile productions, and dominant bacteria.

In our study, the microbial community changed dynamically during storage; the dominant genera in the fresh samples were *Macrococcus*, *Acinetobacter*, *Moraxella*, *Brucella*, and *Pseudomonas*, which changed to *Acinetobacter*, *Carnobacterium*, *Macrococcus*, *Pseudomonas*, and *Psychrobacter* after 10-day storage. Previous literatures demonstrated that *Acinetobacter*, *Moraxella*, *Pseudomonas*, *Aeromonas*, and other Gram-negative bacteria were frequently observed in freshwater fish (Wang et al., 2017; Li et al., 2018c), which was consistent with our results. *Pseudomonas* and *Acinetobacter* are characterized as SSOs and have been demonstrated to play the major contributory role on fish spoilage (Liu et al., 2018a; Shen et al., 2020). Zhang et al. (2015) and Zhao et al. (2021) reported that the spoilage of freshwater fish was associated with *Pseudomonas*, *Shewanella*, and *Aeromonas*, which have been identified in different environment conditions (air or modified atmosphere packages). However, in this study, the proportion of *Shewanella* in refrigerated sturgeon filets was very low and not constructed in the top 30 abundant genera. Therefore, different freshwater fish species may have different spoilage-related bacteria.

Microbial growth is always accompanied by biochemical changes. Previous studies indicated that the microbial metabolic activities cause fish spoilage by affecting the pH, the degradation of nutrients, the generation of TVB-N, and the production of TBARS and VOCs among others (Zhang et al., 2015; Liu et al., 2018a; Li et al., 2020a,b). In the early days of storage, the pH value showed a decreasing trend, which may have resulted from glycolysis and ATP decomposition and lactic acid and pyrophosphate accumulation in fish muscle (Li et al., 2020b). During storage, protein degradation by microorganisms led to an increase of the pH value. Similar results were also reported by Gui et al. (2014) regarding the refrigerated storage of vacuum-packed sturgeon meat. The SDS-PAGE profiles and the concentration of TCA-soluble peptides indicated that bacterial activities promoted the breakdown of sturgeon proteins and produced plenty of water-soluble, small-molecule protein fragments or extracellular protein. Molly et al. (1997) reported that the degradation process was catalyzed by endogenous cathepsin in the early period of storage and further by microbial peptidases during storage. The degradation of protein by microorganisms caused the changes in fish texture and water holding capacity (Gram et al., 2002; Boziaris and Parlapani, 2017). In this study, the degradation products between 70 and 150 kDa might be produced from myosin degradation (Nie et al., 2014). The bands with molecular weight of 15–20 kDa might be generated by *Aeromonas* (Liu et al., 2018a), while the bands of 25–35 kDa may be produced by the spoilage bacteria of *Pseudomonas* and *Shewanella* (Jia et al., 2019; Li et al., 2020a). Li et al. (2020a) reported that myofibrillar proteins, such as tropomyosin (36 kDa) and actin alpha skeletal muscle (45 kDa), could be degraded by microbial activity, which produced lots of water-soluble small-molecule protein fragments. However, in our study, there was no significant band reduction at 220 kDa (myosin) and 45 kDa (actin), which was inconsistent with the findings of Nie et al. (2014). Li et al. (2020b) and Xu et al. (2015) reported that protein degradation was mainly attributed to bacterial activity. However, whether it is from bacterial communities or endoenzymes, protein degradation could contribute significantly on the final quality attributes like *K*-value and the volatile profiles (Feng et al., 2016; Liu et al., 2018a). It should be noted that different bacteria have different protein-degrading effects and pathways—for instance, Zhuang et al. (2022) reported that *Shewanella putrefaciens* was active in hydrolyzing fish proteins (especially collagens), while *Pseudomonas putida* had potent potentials in utilizing oligopeptides and free amino acids. These bacteria have a complementary role in degrading fish proteins, resulting in fish quality deterioration.

In addition, microbial activities also contribute to the accumulation of Hx and HxR, which correspond to the off-flavors and bitter taste of fish. Liu et al. (2010) reported that the accumulation of Hx in fish flesh reflects the initial stage of autolytic degradation and subsequent bacterial spoilage. Our result indicated that bacteria contributed more to the hydrolysis of HxR other than autolysis. Hong et al. (2017) and Liu et al. (2018a) investigated the catabolism of nucleotides in chill-stored shellfish and bighead carp filets, which also found that the conversion of ATP to IMP might be led by autolysis, while the bacteria played prominent roles in the hydrolysis of HxR. Özogul et al. (2008) illustrated that the rapid increase of the *K*-value was due to the sharp decrease of IMP in the fish flesh. When the sensory score was below 5 and the bacterial counts exceeded 7 log CFU/g (day 8), the *K*-value reached to 86.8%, which exceeded the marginal acceptability of 78–85% (Özogul et al., 2006). However, Gui et al. (2014) noted that the acceptability limit of the *K*-value should depend on the species. In our study, the shelf-life of sturgeon filets was less than 8 days after overall consideration of all parameters. However, on day 8, the TVB-N value only reached 18.29 ± 0.24 mg/100 g, which was lower than the acceptable limit of 20 mg/kg for refrigerated silver carp filets (Li et al., 2018b). Therefore, the species should be considered when the TVB-N content is used for the quality classification assessment of fish products. Along with the degradation of peroxides, alcohols, aldehydes, and ketones were formed, which caused off-flavors in the flesh. In this study, VOCs related to spoilage were mainly produced by dominant bacteria *Acinetobacter* and *Carnobacterium*, and 2-pentanone and 2-heptanone might be formed from fish oxidation (Virginia et al., 2012). However, Feng et al. (2017) and Lou et al. (2021) reported some bacteria that might not be the dominant ones in terms of number in a population but could contribute significantly to the volatile profile. Boziaris and Parlapani (2017) reported that ethanol, acetic acid, butanal, 1-propanol, 2-methyl-1-butanol, 3-methyl-1-butanol, 3-hydroxy-2-butanone, 2-butanone, and ethyl acetate were the commonly identified spoilage markers in aquatic products, which can be used to evaluate the freshness or spoilage of fish.

Furthermore, regarding the metabolic pathways, based on 16S rRNA, PICRUSt2 analyzed the functional properties of the bacterial community to provide further insight into the sturgeon filet spoilage process. Carbohydrate and amino metabolism were the main metabolic pathways in the refrigerated fish meat, which was inconsistent with the result of Li et al. (2019). In our study, carbohydrate metabolism was mainly associated with *Macrococcus*, *Brucella*, *Rhodococcus*, *Moraxella*, and *Kocuria*. However, there was a significant lipid metabolism on day 10, which might be related to the increased number of lipid-degrading bacteria since *Acinetobacter* and *Pseudomonas* showed a strong correlation with lipid metabolism. These microorganisms could secrete enzymes involved in lipolysis to promote the lipolytic activity (Mazhar et al., 2020). The three-level pathway of microbial metabolism indicated that microorganisms used the nutrients to grow by degrading proteins and carbohydrates and then promoted its spoilage metabolic activities—for example, the abundance of metabolic pathways associated with biogenic amines and sulfide formation on day 10 was higher than on day 4, such as phenylalanine, tyrosine, and tryptophan biosynthesis metabolism, which was related to *Pseudomonas* and *Enterobacteriaceae* (Mazhar et al., 2020). The pyruvate metabolism associated with the formation of volatile compounds (such as alcohols, aldehydes, ketones, esters, etc.) and glyoxylate and dicarboxylate metabolism was related to the formation of volatile amines (Li et al., 2019). The metabolic pathways might provide insight into the microbial metabolic activity in certain foods and the processes involved in spoilage. However, there is limited understanding about the enzymes responsible for spoilage. Therefore, the enzymes related to spoilage in the main metabolic pathways need further research.

## Conclusion

In summary, we investigated the underlying microbial metabolic activity and biochemical changes in sturgeon filets during refrigerated storage. Bacteria play an important role in the biochemical changes (protein degradation, nucleotide catabolism, TVB-N and TBARS accumulation, VOC production) associated with the spoilage of sturgeon filets. The VOCs like hexanal, ethyl acetate, 4-methyl-2-pentanone, ethanol, butanal, 1-propanol, isopentyl alcohol, 2-pentanone, 2-heptanone, ethyl propanoate, and propyl sulfide were suggested as potential chemical spoilage markers for freshness/spoilage monitoring in sturgeon filet. The dominant bacteria such as *Acinetobacter*, *Macrococcus*, and *Pseudomonas* contributed to the spoilage of refrigerated sturgeon filets, mainly related to carbohydrate metabolism, amino metabolism, and lipid metabolism. This study provides a comprehensive and deep insight into the mechanism of underlying biochemical changes of sturgeon flesh during refrigerated storage. It is important to prevent sturgeon meat spoilage and extend the shelf-life. Further research needs to explore the enzymes related to spoilage in the main metabolic pathways.

## Data Availability Statement

The datasets presented in this study can be found in online repositories. The names of the repository/repositories and accession number(s) can be found in the article/Supplementary Material.

## Author Contributions

CT and PL designed and performed the experiments and analyzed the data. CT wrote the manuscript. NS contributed to the proofreading of the revised manuscript. CT, MX, and RW provided the experimental samples. NS and PL contributed to the manuscript revision and the financial support of this study. All authors have read and agreed to the published version of the manuscript.

## Conflict of Interest

The authors declare that the research was conducted in the absence of any commercial or financial relationships that could be construed as a potential conflict of interest.

## Publisher’s Note

All claims expressed in this article are solely those of the authors and do not necessarily represent those of their affiliated organizations, or those of the publisher, the editors and the reviewers. Any product that may be evaluated in this article, or claim that may be made by its manufacturer, is not guaranteed or endorsed by the publisher.

## Footnotes

1
http://drive5.com/uparse/

2
http://sourceforge.net/projects/rdp-classifier/

3
http://www.arb-silva.de

4
http://www.mothur.org/wiki/
