# Supplementary material for: Unraveling the Effects of Biochemical Drivers on the Bacterial Communities and Volatile Profiles in Refrigerated Sturgeon Filets at 4°C
Source: Front Microbiol. 2022 Mar 30;13:849236. doi: 10.3389/fmicb.2022.849236 (PMC9006255; doi:10.3389/fmicb.2022.849236)
Supplement: Supplementary file 1 [file Data_Sheet_1.PDF]

Supplementary data

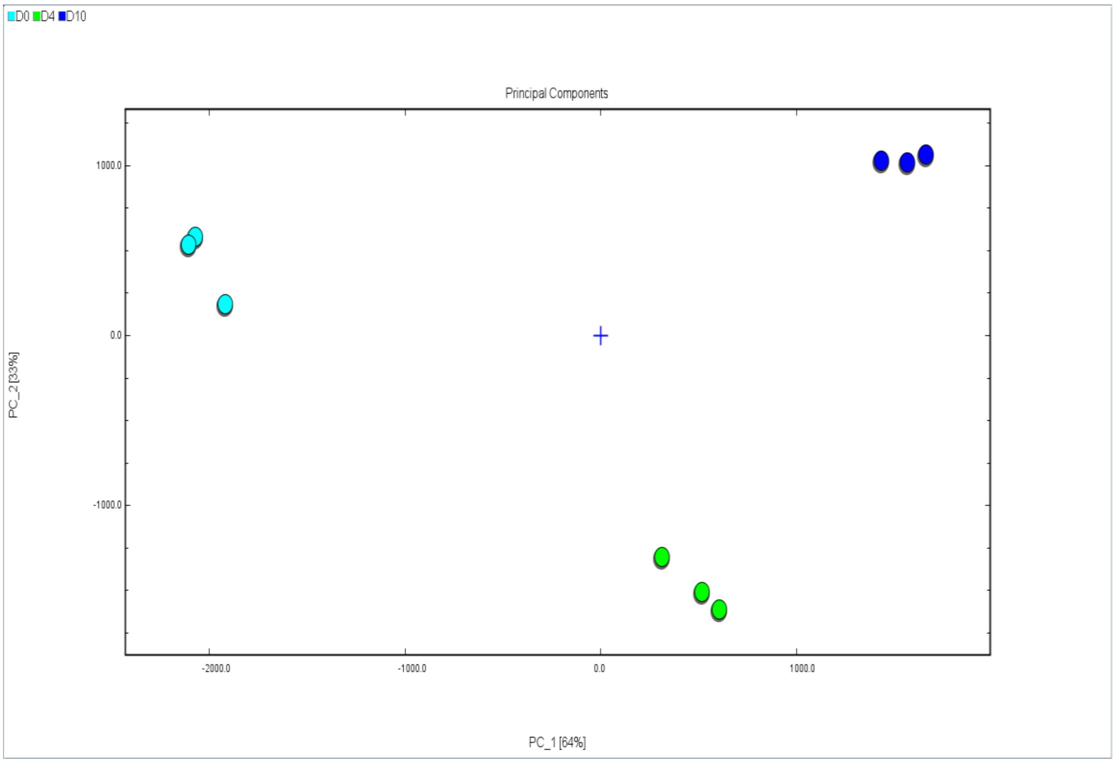

Fig. S1. PCA analysis diagram of VOCs in sturgeon fillets during three refrigeration stages.

Table S1. The information of sequences identified in refrigerated sturgeon samples during storage

Sample Name	Raw PE	Effective Tags	Effective Ratio (%)	Total length	Mean length	Max length	Min length
D0_1	50478	49328	97.72	21631538	428.53	454	250
D0_2	50067	49495	98.86	21399687	427.42	540	250
D0_3	55326	54726	98.92	23708325	428.52	465	250
D4_1	35125	34676	98.72	15066621	428.94	430	271
D4_2	45274	44593	98.50	19414048	428.81	436	271
D4_3	45912	45307	98.68	19687116	428.80	442	281
D10_1	42661	40036	93.85	18336684	429.82	489	345
D10_2	52100	49474	94.96	22395406	429.85	439	277
D10_3	52237	49946	95.61	22456678	429.90	451	313

Table S2. Riches and diversity indices of sturgeon fillets during refrigeration storage

Sample ID	Storage time (day)						
	Effective tags	OTUs	Shannon	Simpson	ACE	Chao1	Coverage/%
D0_1	49328	73	0.798	0.706	103.35	99.54	99.98
D0_2	49495	51	0.822	0.704	82.89	79.40	99.98
D0_3	54726	43	0.592	0.790	65.92	61.77	99.98
Mean_D0	51183±2506	56±13	0.740±0.103	0.730±0.040	84.05±15.30	80.24±15.43	99.98
D4_1	34676	33	0.104	0.971	37.95	64.00	99.98
D4_2	44593	23	0.198	0.937	50.61	41.33	99.98
D4_3	45307	17	0.180	0.946	59.84	53.50	99.97
Mean_D4	41525±4852	24±7	0.160±0.041	0.950±0.014	49.47±8.97	52.94±9.26	99.98
D10_1	40036	33	0.646	0.754	40.97	43.33	99.98
D10_2	49474	30	0.534	0.800	36.46	34.67	99.99
D10_3	49946	29	0.401	0.866	37.61	34.50	99.99
Mean_D10	46485±4564	31±2	0.530±0.100	0.810±0.046	38.35±1.91	37.50±4.12	99.99

Mean values are mean ± standard deviation of three replicates.

Alpha index comparison was calculated by a Student’s t-test and a Kruskal-Wallis H test in R.

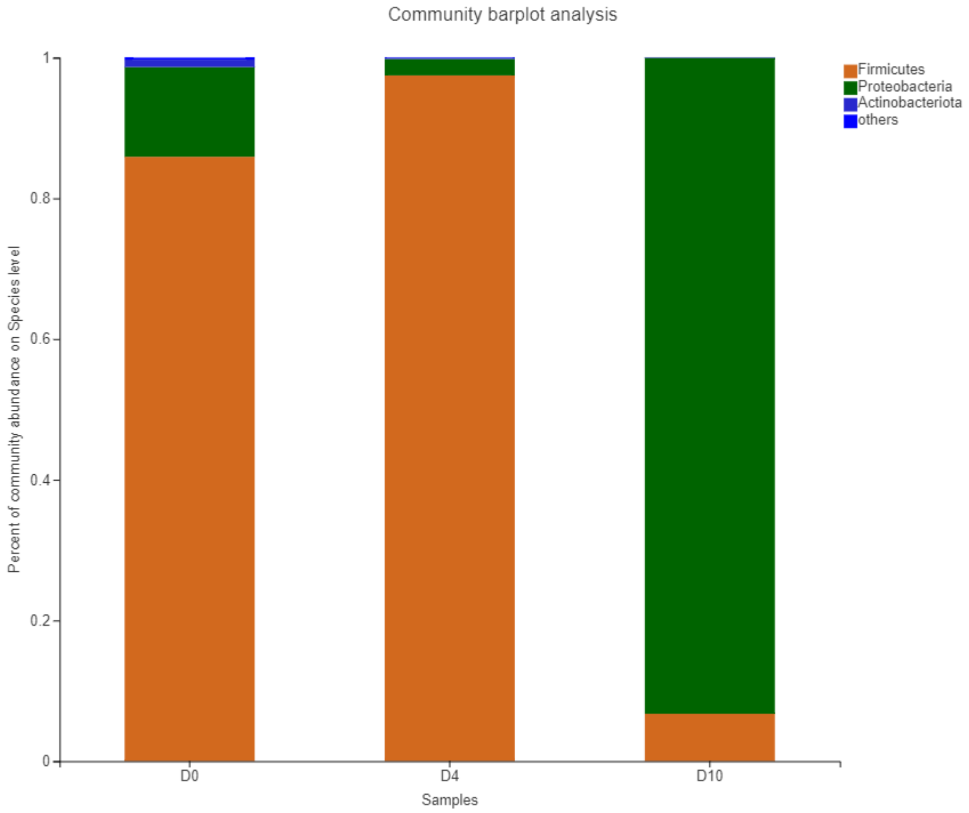

Fig. S2. Community analysis of microbiota at the phylum level of sturgeon samples during storage at 4 °C

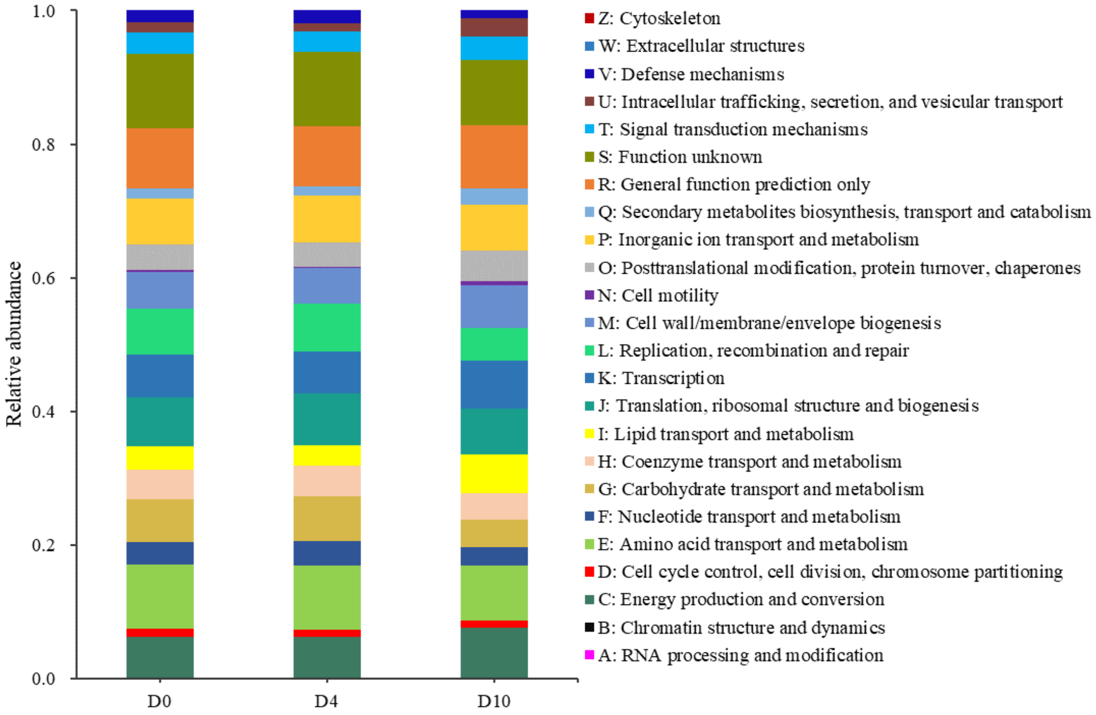

Fig. S3. Changes of microbial metabolic pathways in samples during storage. The number 0, 4 and 10 represent the refrigerated storage time (day).

Table S3. The value of Pearson correlation coefficients (Pearson correlation > |0.7|)

Biochemical indicators	Microbiota	Pearson correlation	*P* value
pH	*Macrococcus*	-0.953983246	6.57887E-05
1_propanol	*Macrococcus*	-0.821822461	0.006573428
2_pentanone_M	*Macrococcus*	-0.821822461	0.006573428
2_pentanone_D	*Macrococcus*	-0.821822461	0.006573428
2_Pentanone_T	*Macrococcus*	-0.821822461	0.006573428
ethyl_propanoate_M	*Macrococcus*	-0.821822461	0.006573428
ethyl_propanoate_D	*Macrococcus*	-0.821822461	0.006573428
2_heptanone_M	*Macrococcus*	-0.821822461	0.006573428
2_heptanone_D	*Macrococcus*	-0.821822461	0.006573428
MTBE	*Acinetobacter*	-0.966240493	2.25207E-05
ethyl_acetate_D	*Acinetobacter*	-0.816666667	0.0107694
Acetoin_M	*Acinetobacter*	-0.91538573	0.000533717
Acetoin_D	*Acinetobacter*	-0.898434142	0.000994195
TVC	*Moraxella*	-0.91538573	0.000533717
TVB_N	*Moraxella*	-0.936246506	0.000202335
Hx	*Moraxella*	-0.961780501	3.46167E-05
HxR	*Moraxella*	-0.936246506	0.000202335
TCA_soluble_peptides	*Moraxella*	-0.864530967	0.002632372
TBARS	*Moraxella*	-0.896551724	0.001058183
Ethanol_M	*Moraxella*	-0.813676204	0.007621974
Ethanol_D	*Moraxella*	-0.949288905	9.20002E-05
1_propanol	*Moraxella*	-0.815730238	0.00734761
butanal	*Moraxella*	-0.949288905	9.20002E-05
2_pentanone_M	*Moraxella*	-0.815730238	0.00734761
2_pentanone_D	*Moraxella*	-0.815730238	0.00734761
2_Pentanone_T	*Moraxella*	-0.815730238	0.00734761
ethyl_propanoate_M	*Moraxella*	-0.815730238	0.00734761
ethyl_propanoate_D	*Moraxella*	-0.815730238	0.00734761
Isopentyl_alcohol_M	*Moraxella*	-0.948275862	9.84961E-05
Isopentyl_alcohol_D	*Moraxella*	-0.965517241	2.42378E-05
2_heptanone_M	*Moraxella*	-0.815730238	0.00734761
2_heptanone_D	*Moraxella*	-0.815730238	0.00734761
Propylsulfide	*Moraxella*	-0.898434142	0.000994195
Appearance	*Carnobacterium*	-0.775862069	0.013986488
Texture	*Carnobacterium*	-0.775862069	0.013986488
Odor	*Carnobacterium*	-0.931034483	0.000265011
IMP	*Carnobacterium*	-0.91538573	0.000533717
ATP	*Carnobacterium*	-0.834110523	0.005184412
ADP	*Carnobacterium*	-0.931657962	0.00025688
AMP	*Carnobacterium*	-0.868155851	0.002402854
ethyl_acetate_D	*Carnobacterium*	-0.711966679	0.031425256
Acetoin_M	*Carnobacterium*	-0.672413793	0.047220088

4_methyl_2_pentanone	*Carnobacterium*	-0.674739827	0.046174853
hexanal_M	*Carnobacterium*	-0.810344828	0.008081599
hexanal_D	*Carnobacterium*	-0.810344828	0.008081599
TVC	*Brucella*	-0.883333333	0.003075397
TVB_N	*Brucella*	-0.903773601	0.000827411
Hx	*Brucella*	-0.828459135	0.005795373
HxR	*Brucella*	-0.853563957	0.003418215
TCA_soluble_peptides	*Brucella*	-0.916666667	0.001311728
TBARS	*Brucella*	-0.949288905	9.20002E-05
Ethanol_D	*Brucella*	-0.883333333	0.003075397
1_propanol	*Brucella*	-0.762413608	0.016908101
butanal	*Brucella*	-0.85	0.006073633
2_pentanone_M	*Brucella*	-0.821822461	0.006573428
2_pentanone_D	*Brucella*	-0.821822461	0.006573428
2_Pentanone_T	*Brucella*	-0.762413608	0.016908101
ethyl_propanoate_M	*Brucella*	-0.762413608	0.016908101
ethyl_propanoate_D	*Brucella*	-0.762413608	0.016908101
Isopentyl_alcohol_M	*Brucella*	-0.864530967	0.002632372
Isopentyl_alcohol_D	*Brucella*	-0.84757938	0.003908548
2_heptanone_M	*Brucella*	-0.762413608	0.016908101
2_heptanone_D	*Brucella*	-0.762413608	0.016908101
Odor	*Psychrobacter*	-0.711966679	0.031425256
IMP	*Psychrobacter*	-0.733333333	0.031123236
ATP	*Psychrobacter*	-0.761512942	0.017117126
ADP	*Psychrobacter*	-0.689099962	0.04004995
AMP	*Psychrobacter*	-0.677830201	0.044809263
4_methyl_2_pentanone	*Psychrobacter*	-0.821822461	0.006573428
hexanal_T	*Psychrobacter*	-0.762413608	0.016908101
MTBE	*Pseudomonas*	-0.745869854	0.021026303
ethyl_acetate_D	*Pseudomonas*	-0.75	0.02549052
Acetoin_M	*Pseudomonas*	-0.779773029	0.013205187
Acetoin_D	*Pseudomonas*	-0.813676204	0.007621974
MTBE	*Macrococcus*	0.864530967	0.002632372
ethyl_acetate_D	*Macrococcus*	0.933333333	0.000749559
Acetoin_M	*Macrococcus*	0.966240493	2.25207E-05
Acetoin_D	*Macrococcus*	0.98319208	1.99397E-06
pH	*Acinetobacter*	0.92051015	0.0004311
1_propanol	*Acinetobacter*	0.821822461	0.006573428
2_pentanone_M	*Acinetobacter*	0.821822461	0.006573428
2_pentanone_D	*Acinetobacter*	0.821822461	0.006573428
2_Pentanone_T	*Acinetobacter*	0.821822461	0.006573428
ethyl_propanoate_M	*Acinetobacter*	0.821822461	0.006573428
ethyl_propanoate_D	*Acinetobacter*	0.821822461	0.006573428
2_heptanone_M	*Acinetobacter*	0.821822461	0.006573428

2_heptanone_D	*Acinetobacter*	0.821822461	0.006573428
Appearance	*Moraxella*	0.948275862	9.84961E-05
Texture	*Moraxella*	0.948275862	9.84961E-05
Odor	*Moraxella*	0.913793103	0.000568803
IMP	*Moraxella*	0.91538573	0.000533717
ATP	*Moraxella*	0.961780501	3.46167E-05
ADP	*Moraxella*	0.906015999	0.00076359
AMP	*Moraxella*	0.91071251	0.000641193
4_methyl_2_pentanone	*Moraxella*	0.795588751	0.010343978
hexanal_M	*Moraxella*	0.896551724	0.001058183
hexanal_D	*Moraxella*	0.896551724	0.001058183
hexanal_T	*Moraxella*	0.795588751	0.010343978
TVC	*Carnobacterium*	0.728918266	0.025883597
TVB_N	*Carnobacterium*	0.834110523	0.005184412
Hx	*Carnobacterium*	0.68090655	0.043475898
HxR	*Carnobacterium*	0.697929213	0.036558747
TCA_soluble_peptides	*Carnobacterium*	0.830627792	0.005555424
TBARS	*Carnobacterium*	0.896551724	0.001058183
Ethanol_D	*Carnobacterium*	0.762821442	0.016814014
1_propanol	*Carnobacterium*	0.775447263	0.014071126
butanal	*Carnobacterium*	0.695015091	0.037688292
2_pentanone_M	*Carnobacterium*	0.835871726	0.005003404
2_pentanone_D	*Carnobacterium*	0.835871726	0.005003404
2_Pentanone_T	*Carnobacterium*	0.775447263	0.014071126
ethyl_propanoate_M	*Carnobacterium*	0.775447263	0.014071126
ethyl_propanoate_D	*Carnobacterium*	0.775447263	0.014071126
Isopentyl_alcohol_M	*Carnobacterium*	0.724137931	0.027375411
Isopentyl_alcohol_D	*Carnobacterium*	0.74137931	0.022248563
2_heptanone_M	*Carnobacterium*	0.775447263	0.014071126
2_heptanone_D	*Carnobacterium*	0.775447263	0.014071126
Appearance	*Brucella*	0.91538573	0.000533717
Texture	*Brucella*	0.91538573	0.000533717
Odor	*Brucella*	0.966240493	2.25207E-05
IMP	*Brucella*	0.933333333	0.000749559
ATP	*Brucella*	0.895405327	0.001098526
ADP	*Brucella*	0.949613362	8.99854E-05
AMP	*Brucella*	0.945614972	0.000117091
ethyl_acetate_M	*Brucella*	0.75	0.02549052
4_methyl_2_pentanone	*Brucella*	0.821822461	0.006573428
hexanal_M	*Brucella*	0.949288905	9.20002E-05
hexanal_D	*Brucella*	0.949288905	9.20002E-05
hexanal_T	*Brucella*	0.821822461	0.006573428
3_methylbutanal_T	*Psychrobacter*	0.766666667	0.021389991
pH	*Pseudomonas*	0.77824949	0.013505973
